# A simple index of lipid overaccumulation is a good marker of liver steatosis

**DOI:** 10.1186/1471-230X-10-98

**Published:** 2010-08-25

**Authors:** Giorgio Bedogni, Henry S Kahn, Stefano Bellentani, Claudio Tiribelli

**Affiliations:** 1 University of Trieste and Liver Research Center, Building Q, AREA Science Park, Strada Statale 14/km 163.5, 34012 Basovizza, Trieste, Italy; 2 Department of Maternal and Pediatric Sciences, University of Milan, Fondazione IRCCS Cà Granda - Ospedale Maggiore Policlinico, Milano, Italy; 3 National Center for Chronic Disease Prevention and Health Promotion, Atlanta, USA; 4 Liver Center, Azienda USL Modena, "Ramazzini" Hospital, Carpi, Modena, Italy

## Abstract

**Background:**

Liver steatosis is often found in association with common cardiometabolic disorders, conditions that may all occur in a shared context of abdominal obesity and dyslipidemia. An algorithm for identifying liver steatosis is the fatty liver index (FLI). The lipid accumulation product (LAP) is an index formulated in a representative sample of the US population to identify cardiometabolic disorders. Because FLI and LAP share two components, namely waist circumference and fasting triglycerides, we evaluated the ability of LAP to identify liver steatosis in the same study population from the Northern Italian town where FLI was initially developed.

**Methods:**

We studied 588 individuals (59% males) aged 21 to 79 years. Liver steatosis was detected by ultrasonography and coded ordinally as none, intermediate and severe. 44% of the individuals had liver steatosis. Using proportional-odds ordinal logistic regression, we evaluated the ability of log-transformed LAP (lnLAP) to identify liver steatosis. We considered the benefits to our model of including terms for sex, age, suspected liver disease and ethanol intake. We calculated the 3-level probability of liver steatosis according to lnLAP and sex, providing tables and nomograms for risk assessment.

**Results:**

An ordinal proportional-odds model consisting of lnLAP and sex offered a reasonably accurate identification of liver steatosis. The odds of more severe *vs. *less severe steatosis increased for increasing values of lnLAP (odds ratio [OR] = 4.28, 95%CI 3.28 to 5.58 for each log-unit increment) and was more likely among males (OR = 1.88, 95%CI 1.31 to 2.69).

**Conclusion:**

In a study sample of adults from Northern Italy, the simple calculation of LAP was a reasonably accurate approach to recognizing individuals with ultrasonographic liver steatosis. LAP may help primary care physicians to select subjects for liver ultrasonography and intensified lifestyle counseling, and researchers to select patients for epidemiologic studies. A more thorough assessment of LAP's potential for identifying liver steatosis will require its cross-evaluation in external populations.

## Background

Fatty liver is rapidly becoming the most common liver disorder worldwide [1-4]. Many individuals with fatty liver have also obesity, type 2 diabetes mellitus and hypertriglyceridemia in the absence of relevant alcohol intake, and they have multiorgan insulin resistance as a hallmark feature [2,5]. Such variety of fatty liver is known as non-alcoholic fatty liver disease (NAFLD) and affects 20-30% of adults in the general population [3]. About 2-3% of the same population is estimated to have non-alcoholic steatohepatitis (NASH), which is a stage of NAFLD that may progress to liver cirrhosis [1,2]. Conventional descriptions of NAFLD depend on a defined threshold estimate of fat content (>5%), but steatosis in the liver is a graded phenomenon that is monotonically associated with some physiologic impairments [1,5]. From a public health perspective, irrespective of its binary (yes *vs. *no) or continuous definition, it has become clear that NAFLD is associated with prevalent and incident cardiovascular disease and diabetes [6-8]. Although it is not yet clear whether NAFLD has a causative role in this respect, there are practical implications for the management of patients with NAFLD [9,10].

With the aim of facilitating the detection of NAFLD in the general population, we took advantage of the Dionysos Nutrition and Liver Study [11], performed in a town of Northern Italy, to develop the "fatty liver index" (FLI). FLI is a continuous measure that identifies the binary condition of fatty liver [12]. FLI is based on body mass index (BMI), waist circumference (WC), fasting triglycerides, and gamma-glutamyl-transferase (GGT). Interestingly, the Relationship between Insulin Sensitivity and Cardiovascular disease risk (RISC) Study has shown that FLI is associated also with insulin resistance, coronary heart disease, and early atherosclerosis in a large European population [13]. FLI has also recently been shown to be a predictor of 9-year incident diabetes in the French Data from an Epidemiological Study on the Insulin Resistance Syndrome (D.E.S.I.R.) Study [14].

One of us has used population-based data from a United States' National Health and Nutrition Examination Survey (NHANES III) to propose a "lipid accumulation product" (LAP) that depends only on the measurement of WC and fasting triglycerides [15]. Cross-sectional analysis within NHANES III demonstrated that LAP was superior to BMI in detecting some prevalent cardiometabolic risk factors and diabetes [15,16]. LAP predicts all-cause mortality [17], and is presently being evaluated for its ability to predict incident cardiometabolic disease.

Because FLI and LAP share two predictors, namely WC and fasting triglycerides, we considered it useful to evaluate the ability of LAP, developed on a representative sample of the US population [15], for detecting liver steatosis in the same Northern Italian study population where FLI was developed [12]. Because the binary classification of liver steatosis into fatty liver (yes *vs. *no) has the potential for losing information on a condition that may be a graded cardiometabolic risk factor [5,18], we evaluated liver steatosis as an ordinal 3-level variable.

## Methods

### Study design

The protocol of the Dionysos Nutrition and Liver Study which is part of the larger Dionysos Project [19-21] and was performed in 2001-2002, is described in detail elsewhere [11]. Briefly, of the 5780 adult residents of Campogalliano (Modena, Emilia-Romagna, Italy), 3345 (58%) agreed to participate to the study and 3329 (99%) of them who had complete demographic data were considered for further evaluation. 497 (15%) of these 3329 participants had suspected liver disease (SLD) according to at least one of the following criteria: 1) alanine transaminase (ALT) > 30 U/L; 2) GGT > 35 U/L; 3) presence of hepatitis B (HBV) surface antigen (HbsAg); 4) presence of hepatitis C virus (HCV) ribonucleic acid (RNA) after detection of anti-HCV antibodies.

With the aim of enriching the analytic sample with persons likely to have liver steatosis, we matched the 497 individuals who met the SLD criteria with an equal number of individuals who had none of the SLD criteria. The individuals without SLD were randomly selected among the remaining 2832 participants with 1:1 matching by sex and age (within 1 year). 324 individuals (65%) in the SLD group and 335 (67%) in the no SLD group agreed to undergo further evaluation and 311 (96%) and 287 (86%) of them had complete data. The present analysis includes 305 participants with SLD and 283 without SLD on the basis of the availability of all measurements of interest (*n *= 588).

### Liver ultrasonography

Liver ultrasonography was performed using standardized criteria by the same operator, who was unaware of the clinical and laboratory data of the participants. Hepatic steatosis was quantified with a method very similar to that recently validated by Hamaguchi *et al. *[22]. *Normal liver *was defined as the absence of liver steatosis or other liver abnormalities. *Light steatosis *was defined as the presence of slight "bright liver" or hepatorenal echo contrast without intrahepatic vessels blurring and no deep attenuation; *moderate steatosis *as the presence of mild "bright liver" or hepatorenal echo contrast without intrahepatic vessel blurring and with deep attenuation; and *severe steatosis *as diffusely severe "bright liver" or hepatorenal echo contrast, with intrahepatic vessels blurring (no visible borders) and deep attenuation without visibility of the diaphragm. Because light and moderate degrees of steatosis are difficult to distinguish and had relatively small numbers in our sample, we pooled together these degrees into one category which we have called "intermediate steatosis".

### Clinical and laboratory assessment

Anthropometry was performed by two dietitians who had been trained and certified before and during the study. Weight and height were measured using standard procedures [23] and WC was measured midway between the lower rib margin and the iliac crest [24]. BMI was calculated as weight (kg)/height (m)^2^.

LAP was calculated by expressing waist enlargement as the measured WC that exceeded a sex-specific minimum WC value and then multiplying waist enlargement by the concentration of fasting triglycerides [15]:

LAP for men = (WC [cm] - 65) × (triglycerides [mmol/L])

LAP for women = (WC [cm] - 58) × (triglycerides [mmol/L])

A seven-day diary was administered to the study participants by two trained dietitians, who discussed it with the participant when she/he returned it one week later [25]. Daily ethanol intake was calculated as the mean value of ethanol intake over a week.

HbsAg and anti-HCV antibodies were assessed and individuals with anti-HCV antibodies underwent an HCV-RNA assessment to confirm HCV infection. ALT, aspartate transaminase (AST), GGT, glucose, triglycerides, and total cholesterol were measured by standard laboratory methods after an overnight fast [11]. Diabetes mellitus was defined as fasting blood glucose ≥ 126 mg/dL or use of anti-diabetic drugs.

### Ethical approval

The study protocol was approved and supervised by the Scientific Committee of the Liver Research Center (Basovizza, Trieste, Italy); all participants gave written informed consent.

### Statistical analysis

Descriptive statistics are reported as 25^th^, 50^th ^and 75^th ^percentiles because of skewed distributions. The 3-level outcome variable, hepatic steatosis assessed by liver ultrasonography, was designated ordinally as none, intermediate and severe. The Jonckheere-Terpstra test for ordered alternatives (both ascending and descending) was used to test the existence of a trend between ordinally coded liver steatosis and LAP and the other variables of interest [26]. Fisher's exact test was used to evaluate the association between categorical variables and liver steatosis [26].

A natural log-transformation of LAP (lnLAP) was performed to ensure the equality of slopes among the levels of the response variable, which is the basic assumption made by the proportional-odds logistic model. We used proportional-odds logistic regression to evaluate the ability of lnLAP to predict liver steatosis, and we considered also alternative pre-specified models that included covariate terms for sex (male *vs. *female), age (years), SLD (yes *vs. *no) and ethanol intake (g/day). The odds ratio (OR) obtained from these models is a measure of the change in the odds from less severe to more severe steatosis [27,28]. The main reason why we took sex, age and SLD into account is that the Dionysos Nutrition and Liver Study is a cross-sectional study with matching of subjects performed on the basis of SLD, age and sex [11].

The equality of slopes among the levels of liver steatosis was checked using the Brant test. Model fit was also evaluated using standard diagnostic plots and the Hosmer-Lemeshow statistic for the 2 binary models underlying the proportional-odds model, i.e. none *vs. *intermediate and severe steatosis and none and intermediate *vs. *severe steatosis. The areas under the receiver-operating characteristic curves (AUROC) corresponding to these models were also calculated as tests of model fit. We compared alternative models using the Bayesian information criterion (BIC). When comparing two models, "weak evidence" in favor of the model with the lower BIC is said to exist when the BIC difference (ΔBIC) is ≤ 2; "positive evidence" when 6 > ΔBIC > 2; "strong evidence" when 6 ≤ ΔBIC < 10; and "very strong evidence" when ΔBIC > 10 [29,30].

All statistical tests were two-tailed and statistical significance was assigned to a *p*-value < 0.05. Statistical analysis was performed using Stata version 11.0 (StataCorp, College Station, TX, USA).

## Results

Table 1 reports the demographic, anthropometric, and metabolic characteristics of the 588 participants (347 males [59%] and 241 females) stratified by the degree of ultrasonographic liver steatosis. Owing to the study design, approximately half (52%, *n *= 305) of the analytic sample had suspected liver disease (SLD). Overall, 256 (44%) of the study participants had intermediate or severe steatosis.

**Table 1:** Measurements of the 588 study subjects.

	None *n *= 332, M = 167, F = 165			Intermediate *n *= 118, M = 79, F = 39			Severe *n *= 138, M = 101, F = 37			JT test
	p50	p25	p75	p50	p25	p75	p50	p25	p75	*p*-value
Age (years)	58	45	69	57	45	64	60	50	65	0.7
Ethanol (g/day)	9	0	27	9	0	28	16	2	43	0.006
Weight (kg)	69.5	61.5	76.5	78.7	71.0	89.0	83.4	75.5	93.2	<0.001
Height (m)	1.64	1.56	1.71	1.67	1.60	1.73	1.66	1.58	1.72	0.003
BMI (kg/m^2^)	25.7	23.8	28.1	28.2	26.0	30.9	30.3	27.9	34.2	<0.001
Waist circumference (cm)	86.5	79.5	93.5	94.5	88.8	102.0	100.8	94.0	109.5	<0.001
ALT (U/L)	19	14	31	26	17	39	29	22	45	<0.001
AST (U/L)	21	17	26	21	18	28	24	20	30	<0.001
GGT (U/L)	18	13	27	27	17	43	36	23	61	<0.001
Glucose (mg/dl)	89	84	97	94	87	102	98	89	110	<0.001
Triglycerides (mg/dl)	90	65	123	115	88	162	149	98	205	<0.001
Total cholesterol (mg/dl)	211	183	236	212	184	238	216	184	244	0.2
LAP	24	15	39	43	27	62	63	36	93	<0.001
lnLAP	3.2	2.7	3.7	3.8	3.3	4.1	4.1	3.6	4.5	<0.001

Abbreviations: M = male; F = female; p50 = 50^th ^percentile (median); p25 = 25^th ^percentile (lower quartile); p75 = 75^th ^percentile (upper quartile); JT test = Jonckerheere-Terpstra test for ordered alternatives (both ascending and descending); BMI = body mass index; ALT = alanine transaminase; AST = aspartate transaminase; GGT = gamma-glutamyl-transferase; LAP = lipid accumulation product; lnLAP = natural logarithm of lipid accumulation product.

56% of the individuals (*n *= 332) had normal liver while 20% (*n *= 118) had intermediate steatosis and 24% (*n *= 138) had severe steatosis. Liver steatosis was more common in males (none = 48%, intermediate = 23%, severe = 29%) than in females (none = 69%, intermediate = 16%, severe = 15%; *p *< 0.001). The distribution of liver steatosis in anti-HbsAg-positive group (*n *= 23) was: none = 19, intermediate = 1, severe = 3; the corresponding numbers for HCV-RNA-positive group (*n *= 60) were 42, 12, 6.

Weight, BMI, WC, ALT, AST, GGT, ethanol intake, glucose, triglycerides and LAP showed an increasing trend for increasing degree of liver steatosis (*p *≤ 0.006). 33 participants had diabetes and 8 of these had normal liver, 8 intermediate steatosis and 17 severe steatosis. The median (25^th ^percentile, 75^th ^percentile) values of LAP in 173 subjects aged 25 to 49 years and in 407 aged 50+ years were 26 (16, 47) and 37 (23, 63) as compared to NHANES III population estimates of 30 (16, 57) and 53 (31, 85) [15].

Table 2 reports the proportional-odds logistic models used to evaluate the association between lnLAP and liver steatosis. Because the Dionysos Nutrition and Liver Study is a cross-sectional study with matching of subjects performed on the basis of SLD, sex and age [11], we tested whether the addition of these variables had any effect on the ability of lnLAP to identify liver steatosis.

**Table 2:** Proportional-odds logistic regression models.

	Model 1 OR [95%CI] *p*-value	Model 2 OR [95%CI] *p*-value	Model 3 OR [95%CI] *p*-value	Model 4 OR [95%CI] *p*-value	Model 5 OR [95%CI] *p*-value
	4.45	4.42	4.28	4.25	4.14
lnLAP	[3.42 to 5.79]	[3.36 to 5.80]	[3.28 to 5.58]	[3.24 to 5.58]	[3.17 to 5.40] *p *< 0.001
	*p *< 0.001	*p *< 0.001	*p *< 0.001	*p *< 0.001	
Male sex	--	1.78	1.88	1.72	1.79
		[1.23 to 2.57]	[1.31 to 2.69]	[1.18 to 2.49]	[1.25 to 2.58]
		*p *= 0.002	*p *< 0.001	*p *= 0.004	*p *= 0.011
Age (years)	--	0.99	--	0.99	--
		[0.98 to 1.01]		[0.98 to 1.01]	
		*p *= 0.232		*p *= 0.316	
SLD	--2;	--	--	1.70	1.72
				[1.20 to 2.41]	[1.21 to 2.44]
				*p *= 0.003	*p *< 0.001
*n*	588	588	588	588	588
*p*-Brant test	0.560	0.518	0.704	0.273	0.331
*p*-HL none *vs. *intermediate + severe steatosis	0.021	0.402	0.836	0.584	0.315
*p*-HL none + intermediate *vs. *severe steatosis	0.236	0.383	0.398	0.594	0.676
AUROC	0.78	0.79	0.79	0.80	0.79
none *vs. *(intermediate + severe) steatosis	[0.74 to 0.82]	[0.76 to 0.83]	[0.76 to 0.83]	[0.76 to 0.83]	[0.76 to 0.83]
AUROC	0.78	0.79	0.79	0.80	0.80
(none + intermediate) *vs. *severe steatosis	[0.74 to 0.82]	[0.75 to 0.83]	[0.76 to 0.83]	[0.76 to 0.84]	[0.76 to 0.84]
BIC	1015	1014	1009	1012	1007

Abbreviations: OR = odds ratio; 95%CI = 95% confidence intervals; *p*-value = *p*-value for the regression coefficient (exponentiated and presented as odds ratio); lnLAP = natural logarithm of the lipid accumulation product; SLD = suspected liver disease; *p*-Brant = *p*-value associated with the Brant test of proportional odds; *p*-HL = *p*-value associated with the Hosmer Lemeshow statistics of the 2 binary models underlying the proportional-odds model; AUROC = area under the ROC curve; BIC = Bayesian information criterion.

*Model 1 *shows that for every increase in 1 unit of lnLAP, the odds of more severe *vs. *less severe liver steatosis was 4.45 (95%CI 3.42 to 5.79, *p *< 0.001). However, the fit of Model 1 was not good, as detected by the Hosmer-Lemeshow statistic for the binary logistic model aiming to discriminate none *vs. *intermediate and severe liver steatosis (*p *= 0.008). *Model 2 *added sex and age to lnLAP and showed an independent effect of sex but not of age on liver steatosis. *Model 3 *removed the non-significant age term from Model 2 and offered "strong evidence" of improvement as compared to Model 1 (ΔBIC = -6). Model 3 also fitted well according to the Hosmer-Lemeshow statistics. *Model 4 *added SLD to the predictors of Model 2. As the effect of age was still not significant in Model 4, *Model 5 *evaluated the degree to which the addition of SLD ameliorated the fit of *Model 3*. There was only "weak evidence" of improvement of *Model 5 vs. Model 3 *(ΔBIC = -2), which is clearly not counterbalanced by the difficulties in evaluating SLD in epidemiological studies outside the field of hepatology. The AUROC were similar for all models suggesting no advantage in using the more complex models. However, the AUROC does not address the issue of model calibration so that it must be interpreted in the light of the results of the other tests [31].

Because ethanol intake was higher in males than in females, we tested whether it could be partly responsible for the sex-related difference in the liver steatosis-lnLAP association by adding it (g/day) to *Models 1-5 *but found no association between it and liver steatosis (data not shown). This finding was not unexpected owing to our previous demonstration that alcohol intake was not a predictor of binary fatty liver in this population [12] and with the independent observation made by the RISC Study that FLI and alcohol intake are not associated [13].

Another possible explanation for the sex-related difference in the liver steatosis-lnLAP association could be that we measured WC at the midpoint between the last rib and the iliac crest [11,24] while LAP was developed from the NHANES data using waist measured at level of the iliac crest [15,32]. While these alternative WC measurement protocols provide similar values in men, in women the iliac-crest site may overestimate WC by about 1.8 cm as compared to the site midway between the last rib and iliac crest [33]. We tried to take into account this difference by subtracting 1.8 cm from the WC of our women and refitting *Models 1-5 *(data not shown). The results were virtually unchanged as compared to those provided in Table 2. This was not unexpected because the relationship between WC and health outcomes is fairly stable independently from the measurement site [34].

**Figure 1:**
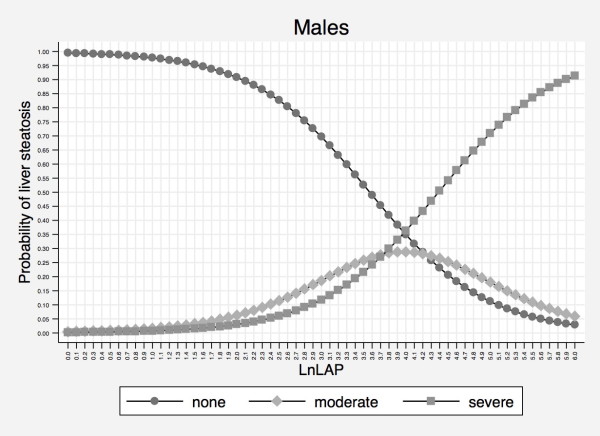
**Probability of liver steatosis as detected by the natural logarithm of the lipid accumulation product in males**. Abbreviations: lnLAP = natural logarithm of LAP.

**Figure 2:**
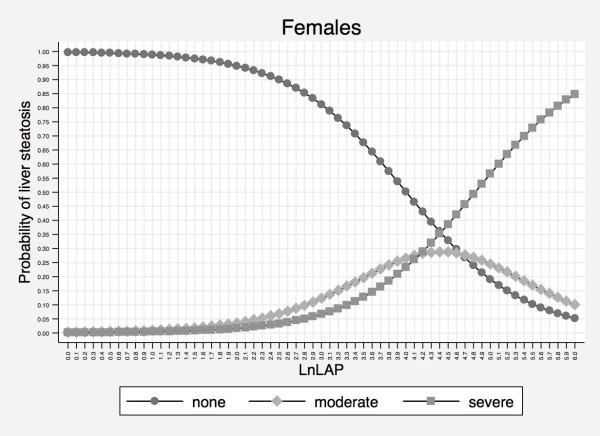
**Probability of liver steatosis as detected by the natural logarithm of the lipid accumulation product in females**. Abbreviations: lnLAP = natural logarithm of LAP.

Owing to this evidence, we choose *Model 3*, based on lnLAP and sex, as the most efficient and practical model for predicting liver steatosis in our study population. Figures 1 and 2 displays sex-specific probabilities of 3-level liver steatosis as nomograms that illustrate the continuous relationship of liver steatosis to lnLAP. The Additional file 1 reports the probability of 3-level liver steatosis for increments of 0.1 units in lnLAP in separate tables for males and females.

## Discussion

We evaluated whether LAP, developed as an index of cardiometabolic risk in a representative sample of the US population [15], could be employed as a predictor of liver steatosis in the same Northern Italian study population that was used to develop FLI [12]. We were prompted to perform this evaluation because FLI, originally derived to identify persons with fatty liver, has recently been shown to identify cardiometabolic risk by the RISC Study [13] and incident diabetes by the D.E.S.I.R. Study [14]; FLI shares with LAP 2 of its 4 components, namely WC and serum triglycerides.

We found that increasing values of LAP are associated with increasing degrees of liver steatosis and that sex has to be considered in this relationship. Although the discriminative ability of lnLAP with respect to liver steatosis was only modestly affected by sex, the inclusion of sex in our ordinal logistic model improved model calibration (Table 2). While the curves depicting the probability of liver steatosis as a function of lnLAP have a similar shape in males and females, they tend to be shifted to the right in the latter, indicating that males have a higher probability of more severe steatosis at the same value of LnLAP (Figures 1 and 2).

Our study has some limitations. First, although LAP has the benefit of having been derived from a sample that represented the US population (NHANES III) [15], it has not been evaluated for the prediction of liver steatosis in the same population. Thus, strictly speaking, our study is not an external "validation" but an external "evaluation" of LAP as simple index of liver steatosis. Second, although the Dionysos Nutrition and Liver Study was performed in a sample drawn from the general adult population of a town in Northern Italy, this population is not representative of Italy as a whole [11,12]. LAP was independent from sex in the original analysis [15] and the sex-lnLAP relationship observed in the present study may be explained not only by the different outcome variable (liver steatosis *vs. *cardiovascular risk factors) but also by local characteristics of the population studied. Third, even if ultrasonography is at present the most practical option to detect liver steatosis in epidemiological studies, it underestimates the prevalence of fatty liver [2,3] and, more importantly, does not offer information on the presence of NASH and liver fibrosis [1-3]. However, in a clinical series of children with NAFLD, we have recently reported that WC and triglycerides can be used to predict the presence of liver fibrosis, which is an "hard" hepatologic outcome [35]. Fourth, it remains to be tested whether the addition of BMI and GGT to LAP - equivalent to testing all four of the components which make up FLI - can improve the identification of liver steatosis sufficiently to justify the monetary and non-monetary costs associated with these additional two components. Such a comparison should performed in external populations because testing FLI in the same population in which it was developed is expected to overestimate its accuracy [36]. Fifth, the ability of lnLAP to discriminate between intermediate and severe steatosis is however limited up to values of 4.0 in males and 4.4 females (Figures 1 and 2). Whether this is a true limitation of LAP will require epidemiological studies trying to answer the more fundamental question whether the fat content of the liver is associated with hard clinical outcomes as it is for some physiological outcomes [5].

The association between LAP, liver steatosis and cardiometabolic disease [15,16] might be partially explained by a common pathophysiological milieu. Besides being a recognized risk factor for cardiovascular disease [34], waist circumference is a surrogate measure of visceral fat, which is the most abundant form of ectopic fat and which is thought to play a major role in insulin resistance and lipotoxicity [37,38]. Serum triglycerides are commonly elevated in the presence of insulin resistance and hyperlipidemia, a recognized risk factor for cardiovascular disease, is strongly associated with hepatic triacyglicerol content [6]. The existence of a liver-vessel axis has been recently hypothesized to explain the association between NAFLD and cardiovascular disease [6]. Moreover, recent publications suggest that steatosis in the liver may be the first [39] or the best [40] marker associated with cardiometabolic risk. LAP may thus be valuable for recognizing patients likely to have insulin resistance along with ectopic lipid deposition also in non-hepatic tissues [41].

## Conclusion

LAP, developed as a marker of cardiometabolic risk in a representative sample of the US population, proved also to be a simple and reasonably accurate predictor of ultrasonographic liver steatosis in a study sample derived from the adult population of a town in Northern Italy. LAP may help primary care physicians to select subjects for liver ultrasonography and intensified lifestyle counseling, and researchers to select patients for epidemiologic studies. LAP, a continuous index that can vary independently of body weight [42], may also be useful for the low-cost monitoring of metabolic deterioration or the benefits associated with exercise, diet, behavior therapy or pharmacological treatments. A more thorough test of LAP's potential for identifying liver steatosis and its changes following treatment will require its cross-evaluation in external populations.

## Abbreviations

95%CI: 95% confidence interval; ALT: alanine transaminase; AST: aspartate transaminase; BIC: bayesian information criterion; BMI: body mass index; D.E.S.I.R.: Data from an Epidemiological Study on the Insulin Resistance Syndrome Study; FLI: fatty liver index; GGT: gamma-glutamyl-transferase; HbsAg: hepatitis B surface antigen; HBV: hepatitis B virus; HCV: hepatitis C virus; HCV-RNA: ribonucleic acid of hepatitis C virus; LAP: lipid accumulation product; LnLAP: natural logarithm of lipid accumulation product; NAFLD: non-alcoholic fatty liver disease; NHANES: National Health and Nutrition Examination Survey; OR: odds ratio; RISC: Relationship between Insulin Sensitivity and Cardiovascular disease risk Study; SLD: suspected liver disease; WC: waist circumference.

## Competing interests

The authors declare that they have no competing interests.

## Authors' contributions

GB co-designed the Dionysos Nutrition and Liver Study, performed statistical analysis and drafted the manuscript; HSK conceived the study and helped to draft the manuscript; SB co-designed the Dionysos Nutrition and Liver Study and helped to draft the manuscript; CT co-designed the Dionysos Nutrition and Liver Study and helped to draft the manuscript. All authors read and approved the manuscript.

## Pre-publication history

The pre-publication history for this paper can be accessed here:

http://www.biomedcentral.com/1471-230X/10/98/prepub

## Supplementary Material

Additional file 1 **Probability of liver steatosis as detected by the natural logarithm of the lipid accumulation product in males and females**. Abbreviations; LAP = lipid accumulation product; lnLAP = natural logarithm of LAP; Prob = probability; Lower = lower 95% confidence interval; Upper = upper 95% confidence interval. Click here for file
