# Supplementary material for: A simple index of lipid overaccumulation is a good marker of liver steatosis
Source: BMC Gastroenterol. 2010 Aug 25;10:98. doi: 10.1186/1471-230X-10-98 (PMC2940930; doi:10.1186/1471-230X-10-98)
Supplement: Additional file 1 — Probability of liver steatosis as detected by the natural logarithm of the lipid accumulation product in males and females. Abbreviations; LAP = lipid accumulation product; lnLAP = natural logarithm of LAP; Prob = probability; Lower = lower 95% confidence interval; Upper = upper 95% confidence interval. [file 1471-230X-10-98-S1.DOC]

|  |  | Probability of liver steatosis in MALES | | | | | | | | |
| --- | --- | --- | --- | --- | --- | --- | --- | --- | --- | --- |
|  |  | None | | | Intermediate | | | Severe | | |
| LAP | LnLAP | Prob | Lower | Upper | Prob | Lower | Upper | Prob | Lower | Upper |
| 1.0 | 0.0 | 0.99 | 0.99 | 1.00 | 0.00 | 0.00 | 0.01 | 0.00 | 0.00 | 0.00 |
| 1.1 | 0.1 | 0.99 | 0.99 | 1.00 | 0.00 | 0.00 | 0.01 | 0.00 | 0.00 | 0.00 |
| 1.2 | 0.2 | 0.99 | 0.99 | 1.00 | 0.01 | 0.00 | 0.01 | 0.00 | 0.00 | 0.00 |
| 1.3 | 0.3 | 0.99 | 0.98 | 1.00 | 0.01 | 0.00 | 0.01 | 0.00 | 0.00 | 0.01 |
| 1.5 | 0.4 | 0.99 | 0.98 | 1.00 | 0.01 | 0.00 | 0.01 | 0.00 | 0.00 | 0.01 |
| 1.6 | 0.5 | 0.99 | 0.98 | 1.00 | 0.01 | 0.00 | 0.01 | 0.00 | 0.00 | 0.01 |
| 1.8 | 0.6 | 0.99 | 0.98 | 1.00 | 0.01 | 0.00 | 0.02 | 0.00 | 0.00 | 0.01 |
| 2.0 | 0.7 | 0.98 | 0.97 | 1.00 | 0.01 | 0.00 | 0.02 | 0.00 | 0.00 | 0.01 |
| 2.2 | 0.8 | 0.98 | 0.97 | 1.00 | 0.01 | 0.00 | 0.02 | 0.01 | 0.00 | 0.01 |
| 2.5 | 0.9 | 0.98 | 0.96 | 0.99 | 0.01 | 0.00 | 0.02 | 0.01 | 0.00 | 0.01 |
| 2.7 | 1.0 | 0.98 | 0.96 | 0.99 | 0.02 | 0.00 | 0.03 | 0.01 | 0.00 | 0.01 |
| 3.0 | 1.1 | 0.97 | 0.95 | 0.99 | 0.02 | 0.01 | 0.03 | 0.01 | 0.00 | 0.01 |
| 3.3 | 1.2 | 0.97 | 0.95 | 0.99 | 0.02 | 0.01 | 0.04 | 0.01 | 0.00 | 0.02 |
| 3.7 | 1.3 | 0.96 | 0.94 | 0.99 | 0.02 | 0.01 | 0.04 | 0.01 | 0.00 | 0.02 |
| 4.1 | 1.4 | 0.96 | 0.93 | 0.98 | 0.03 | 0.01 | 0.04 | 0.01 | 0.00 | 0.02 |
| 4.5 | 1.5 | 0.95 | 0.93 | 0.98 | 0.03 | 0.01 | 0.05 | 0.01 | 0.01 | 0.02 |
| 5.0 | 1.6 | 0.95 | 0.92 | 0.98 | 0.04 | 0.02 | 0.06 | 0.02 | 0.01 | 0.03 |
| 5.5 | 1.7 | 0.94 | 0.91 | 0.97 | 0.04 | 0.02 | 0.06 | 0.02 | 0.01 | 0.03 |
| 6.0 | 1.8 | 0.93 | 0.89 | 0.96 | 0.05 | 0.02 | 0.07 | 0.02 | 0.01 | 0.04 |
| 6.7 | 1.9 | 0.92 | 0.88 | 0.96 | 0.05 | 0.03 | 0.08 | 0.03 | 0.01 | 0.04 |
| 7.4 | 2.0 | 0.91 | 0.87 | 0.95 | 0.06 | 0.04 | 0.09 | 0.03 | 0.01 | 0.05 |
| 8.2 | 2.1 | 0.89 | 0.85 | 0.94 | 0.07 | 0.04 | 0.10 | 0.03 | 0.02 | 0.05 |
| 9.0 | 2.2 | 0.88 | 0.83 | 0.93 | 0.08 | 0.05 | 0.11 | 0.04 | 0.02 | 0.06 |
| 10.0 | 2.3 | 0.86 | 0.81 | 0.91 | 0.09 | 0.06 | 0.12 | 0.05 | 0.03 | 0.07 |
| 11.0 | 2.4 | 0.85 | 0.79 | 0.90 | 0.10 | 0.07 | 0.13 | 0.05 | 0.03 | 0.08 |
| 12.2 | 2.5 | 0.83 | 0.77 | 0.88 | 0.11 | 0.08 | 0.15 | 0.06 | 0.04 | 0.08 |
| 13.5 | 2.6 | 0.80 | 0.75 | 0.86 | 0.13 | 0.09 | 0.16 | 0.07 | 0.04 | 0.10 |
| 14.9 | 2.7 | 0.78 | 0.72 | 0.84 | 0.14 | 0.10 | 0.18 | 0.08 | 0.05 | 0.11 |
| 16.4 | 2.8 | 0.75 | 0.69 | 0.81 | 0.16 | 0.12 | 0.19 | 0.09 | 0.06 | 0.12 |
| 18.2 | 2.9 | 0.73 | 0.67 | 0.79 | 0.17 | 0.13 | 0.21 | 0.10 | 0.07 | 0.14 |
| 20.1 | 3.0 | 0.70 | 0.64 | 0.76 | 0.19 | 0.15 | 0.22 | 0.12 | 0.08 | 0.15 |
| 22.2 | 3.1 | 0.66 | 0.60 | 0.73 | 0.20 | 0.16 | 0.24 | 0.13 | 0.10 | 0.17 |
| 24.5 | 3.2 | 0.63 | 0.57 | 0.69 | 0.22 | 0.18 | 0.26 | 0.15 | 0.11 | 0.19 |
| 27.1 | 3.3 | 0.60 | 0.54 | 0.66 | 0.23 | 0.19 | 0.27 | 0.17 | 0.13 | 0.21 |
| 30.0 | 3.4 | 0.56 | 0.50 | 0.62 | 0.25 | 0.20 | 0.29 | 0.19 | 0.15 | 0.23 |
| 33.1 | 3.5 | 0.53 | 0.47 | 0.58 | 0.26 | 0.22 | 0.30 | 0.22 | 0.17 | 0.26 |
| 36.6 | 3.6 | 0.49 | 0.43 | 0.55 | 0.27 | 0.22 | 0.31 | 0.24 | 0.20 | 0.29 |
| 40.4 | 3.7 | 0.45 | 0.40 | 0.51 | 0.28 | 0.23 | 0.32 | 0.27 | 0.22 | 0.32 |
| 44.7 | 3.8 | 0.42 | 0.36 | 0.47 | 0.28 | 0.24 | 0.33 | 0.30 | 0.25 | 0.35 |
| 49.4 | 3.9 | 0.38 | 0.33 | 0.44 | 0.29 | 0.24 | 0.33 | 0.33 | 0.28 | 0.38 |
| 54.6 | 4.0 | 0.35 | 0.29 | 0.41 | 0.29 | 0.24 | 0.33 | 0.36 | 0.31 | 0.42 |
| 60.3 | 4.1 | 0.32 | 0.26 | 0.37 | 0.29 | 0.24 | 0.33 | 0.40 | 0.34 | 0.46 |
| 66.7 | 4.2 | 0.29 | 0.23 | 0.34 | 0.28 | 0.24 | 0.33 | 0.43 | 0.37 | 0.50 |
| 73.7 | 4.3 | 0.26 | 0.20 | 0.31 | 0.27 | 0.23 | 0.32 | 0.47 | 0.40 | 0.54 |
| 81.5 | 4.4 | 0.23 | 0.18 | 0.28 | 0.26 | 0.22 | 0.31 | 0.50 | 0.43 | 0.58 |
| 90.0 | 4.5 | 0.21 | 0.15 | 0.26 | 0.25 | 0.21 | 0.30 | 0.54 | 0.47 | 0.62 |
| 99.5 | 4.6 | 0.18 | 0.13 | 0.23 | 0.24 | 0.19 | 0.29 | 0.58 | 0.50 | 0.65 |
| 109.9 | 4.7 | 0.16 | 0.11 | 0.21 | 0.23 | 0.18 | 0.27 | 0.61 | 0.53 | 0.69 |
| 121.5 | 4.8 | 0.14 | 0.10 | 0.19 | 0.21 | 0.16 | 0.26 | 0.65 | 0.56 | 0.73 |
| 134.3 | 4.9 | 0.13 | 0.08 | 0.17 | 0.19 | 0.15 | 0.24 | 0.68 | 0.60 | 0.76 |
| 148.4 | 5.0 | 0.11 | 0.07 | 0.15 | 0.18 | 0.13 | 0.23 | 0.71 | 0.63 | 0.79 |
| 164.0 | 5.1 | 0.10 | 0.06 | 0.14 | 0.16 | 0.12 | 0.21 | 0.74 | 0.66 | 0.82 |
| 181.3 | 5.2 | 0.09 | 0.05 | 0.12 | 0.15 | 0.10 | 0.20 | 0.77 | 0.69 | 0.84 |
| 200.3 | 5.3 | 0.07 | 0.04 | 0.11 | 0.13 | 0.09 | 0.18 | 0.79 | 0.71 | 0.87 |
| 221.4 | 5.4 | 0.07 | 0.03 | 0.10 | 0.12 | 0.08 | 0.17 | 0.81 | 0.74 | 0.89 |
| 244.7 | 5.5 | 0.06 | 0.03 | 0.09 | 0.11 | 0.06 | 0.15 | 0.83 | 0.76 | 0.90 |
| 270.4 | 5.6 | 0.05 | 0.02 | 0.08 | 0.10 | 0.05 | 0.14 | 0.85 | 0.79 | 0.92 |
| 298.9 | 5.7 | 0.04 | 0.02 | 0.07 | 0.09 | 0.05 | 0.13 | 0.87 | 0.81 | 0.93 |
| 330.3 | 5.8 | 0.04 | 0.02 | 0.06 | 0.08 | 0.04 | 0.11 | 0.89 | 0.83 | 0.94 |
| 365.0 | 5.9 | 0.03 | 0.01 | 0.05 | 0.07 | 0.03 | 0.10 | 0.90 | 0.85 | 0.95 |
| 403.4 | 6.0 | 0.03 | 0.01 | 0.05 | 0.06 | 0.03 | 0.09 | 0.91 | 0.86 | 0.96 |
|  |  | Probability of liver steatosis in FEMALES | | | | | | | | |
|  |  | None | | | Intermediate | | | Severe | | |
| LAP | LnLAP | Prob | Lower | Upper | Prob | Lower | Upper | Prob | Lower | Upper |
| 1.0 | 0.0 | 1.00 | 0.99 | 1.00 | 0.00 | 0.00 | 0.00 | 0.00 | 0.00 | 0.00 |
| 1.1 | 0.1 | 1.00 | 0.99 | 1.00 | 0.00 | 0.00 | 0.00 | 0.00 | 0.00 | 0.00 |
| 1.2 | 0.2 | 1.00 | 0.99 | 1.00 | 0.00 | 0.00 | 0.01 | 0.00 | 0.00 | 0.00 |
| 1.3 | 0.3 | 1.00 | 0.99 | 1.00 | 0.00 | 0.00 | 0.01 | 0.00 | 0.00 | 0.00 |
| 1.5 | 0.4 | 0.99 | 0.99 | 1.00 | 0.00 | 0.00 | 0.01 | 0.00 | 0.00 | 0.00 |
| 1.6 | 0.5 | 0.99 | 0.99 | 1.00 | 0.00 | 0.00 | 0.01 | 0.00 | 0.00 | 0.00 |
| 1.8 | 0.6 | 0.99 | 0.99 | 1.00 | 0.00 | 0.00 | 0.01 | 0.00 | 0.00 | 0.00 |
| 2.0 | 0.7 | 0.99 | 0.99 | 1.00 | 0.01 | 0.00 | 0.01 | 0.00 | 0.00 | 0.00 |
| 2.2 | 0.8 | 0.99 | 0.98 | 1.00 | 0.01 | 0.00 | 0.01 | 0.00 | 0.00 | 0.01 |
| 2.5 | 0.9 | 0.99 | 0.98 | 1.00 | 0.01 | 0.00 | 0.01 | 0.00 | 0.00 | 0.01 |
| 2.7 | 1.0 | 0.99 | 0.98 | 1.00 | 0.01 | 0.00 | 0.01 | 0.00 | 0.00 | 0.01 |
| 3.0 | 1.1 | 0.99 | 0.98 | 1.00 | 0.01 | 0.00 | 0.02 | 0.00 | 0.00 | 0.01 |
| 3.3 | 1.2 | 0.98 | 0.97 | 0.99 | 0.01 | 0.00 | 0.02 | 0.01 | 0.00 | 0.01 |
| 3.7 | 1.3 | 0.98 | 0.97 | 0.99 | 0.01 | 0.00 | 0.02 | 0.01 | 0.00 | 0.01 |
| 4.1 | 1.4 | 0.98 | 0.96 | 0.99 | 0.02 | 0.01 | 0.02 | 0.01 | 0.00 | 0.01 |
| 4.5 | 1.5 | 0.97 | 0.96 | 0.99 | 0.02 | 0.01 | 0.03 | 0.01 | 0.00 | 0.01 |
| 5.0 | 1.6 | 0.97 | 0.95 | 0.99 | 0.02 | 0.01 | 0.03 | 0.01 | 0.00 | 0.02 |
| 5.5 | 1.7 | 0.97 | 0.95 | 0.99 | 0.02 | 0.01 | 0.04 | 0.01 | 0.00 | 0.02 |
| 6.0 | 1.8 | 0.96 | 0.94 | 0.98 | 0.03 | 0.01 | 0.04 | 0.01 | 0.00 | 0.02 |
| 6.7 | 1.9 | 0.96 | 0.93 | 0.98 | 0.03 | 0.02 | 0.05 | 0.01 | 0.01 | 0.02 |
| 7.4 | 2.0 | 0.95 | 0.92 | 0.97 | 0.04 | 0.02 | 0.05 | 0.02 | 0.01 | 0.03 |
| 8.2 | 2.1 | 0.94 | 0.91 | 0.97 | 0.04 | 0.02 | 0.06 | 0.02 | 0.01 | 0.03 |
| 9.0 | 2.2 | 0.93 | 0.90 | 0.96 | 0.05 | 0.03 | 0.07 | 0.02 | 0.01 | 0.03 |
| 10.0 | 2.3 | 0.92 | 0.89 | 0.95 | 0.05 | 0.03 | 0.07 | 0.03 | 0.01 | 0.04 |
| 11.0 | 2.4 | 0.91 | 0.88 | 0.95 | 0.06 | 0.04 | 0.08 | 0.03 | 0.02 | 0.04 |
| 12.2 | 2.5 | 0.90 | 0.86 | 0.94 | 0.07 | 0.04 | 0.09 | 0.03 | 0.02 | 0.05 |
| 13.5 | 2.6 | 0.89 | 0.85 | 0.93 | 0.08 | 0.05 | 0.10 | 0.04 | 0.02 | 0.05 |
| 14.9 | 2.7 | 0.87 | 0.83 | 0.91 | 0.09 | 0.06 | 0.11 | 0.04 | 0.03 | 0.06 |
| 16.4 | 2.8 | 0.85 | 0.81 | 0.90 | 0.10 | 0.07 | 0.13 | 0.05 | 0.03 | 0.07 |
| 18.2 | 2.9 | 0.83 | 0.79 | 0.88 | 0.11 | 0.08 | 0.14 | 0.06 | 0.04 | 0.08 |
| 20.1 | 3.0 | 0.81 | 0.76 | 0.86 | 0.12 | 0.09 | 0.15 | 0.07 | 0.04 | 0.09 |
| 22.2 | 3.1 | 0.79 | 0.74 | 0.84 | 0.14 | 0.10 | 0.17 | 0.08 | 0.05 | 0.10 |
| 24.5 | 3.2 | 0.76 | 0.71 | 0.82 | 0.15 | 0.11 | 0.19 | 0.09 | 0.06 | 0.11 |
| 27.1 | 3.3 | 0.74 | 0.68 | 0.79 | 0.17 | 0.13 | 0.20 | 0.10 | 0.07 | 0.13 |
| 30.0 | 3.4 | 0.71 | 0.65 | 0.77 | 0.18 | 0.14 | 0.22 | 0.11 | 0.08 | 0.15 |
| 33.1 | 3.5 | 0.68 | 0.61 | 0.74 | 0.20 | 0.16 | 0.24 | 0.13 | 0.09 | 0.16 |
| 36.6 | 3.6 | 0.64 | 0.58 | 0.71 | 0.21 | 0.17 | 0.25 | 0.15 | 0.11 | 0.19 |
| 40.4 | 3.7 | 0.61 | 0.54 | 0.68 | 0.23 | 0.18 | 0.27 | 0.16 | 0.12 | 0.21 |
| 44.7 | 3.8 | 0.57 | 0.50 | 0.65 | 0.24 | 0.20 | 0.29 | 0.19 | 0.14 | 0.23 |
| 49.4 | 3.9 | 0.54 | 0.46 | 0.61 | 0.25 | 0.21 | 0.30 | 0.21 | 0.16 | 0.26 |
| 54.6 | 4.0 | 0.50 | 0.42 | 0.58 | 0.27 | 0.22 | 0.31 | 0.23 | 0.17 | 0.29 |
| 60.3 | 4.1 | 0.47 | 0.39 | 0.55 | 0.27 | 0.23 | 0.32 | 0.26 | 0.20 | 0.32 |
| 66.7 | 4.2 | 0.43 | 0.35 | 0.51 | 0.28 | 0.24 | 0.33 | 0.29 | 0.22 | 0.36 |
| 73.7 | 4.3 | 0.39 | 0.31 | 0.48 | 0.29 | 0.24 | 0.33 | 0.32 | 0.24 | 0.40 |
| 81.5 | 4.4 | 0.36 | 0.28 | 0.44 | 0.29 | 0.24 | 0.33 | 0.35 | 0.27 | 0.43 |
| 90.0 | 4.5 | 0.33 | 0.24 | 0.41 | 0.29 | 0.24 | 0.33 | 0.39 | 0.30 | 0.47 |
| 99.5 | 4.6 | 0.30 | 0.21 | 0.38 | 0.28 | 0.24 | 0.33 | 0.42 | 0.33 | 0.52 |
| 109.9 | 4.7 | 0.27 | 0.19 | 0.35 | 0.28 | 0.23 | 0.32 | 0.46 | 0.36 | 0.56 |
| 121.5 | 4.8 | 0.24 | 0.16 | 0.32 | 0.27 | 0.22 | 0.32 | 0.49 | 0.39 | 0.60 |
| 134.3 | 4.9 | 0.21 | 0.14 | 0.29 | 0.26 | 0.20 | 0.31 | 0.53 | 0.42 | 0.64 |
| 148.4 | 5.0 | 0.19 | 0.12 | 0.26 | 0.24 | 0.19 | 0.30 | 0.57 | 0.45 | 0.68 |
| 164.0 | 5.1 | 0.17 | 0.10 | 0.24 | 0.23 | 0.17 | 0.29 | 0.60 | 0.49 | 0.71 |
| 181.3 | 5.2 | 0.15 | 0.08 | 0.22 | 0.22 | 0.16 | 0.28 | 0.63 | 0.52 | 0.75 |
| 200.3 | 5.3 | 0.13 | 0.07 | 0.19 | 0.20 | 0.14 | 0.26 | 0.67 | 0.55 | 0.78 |
| 221.4 | 5.4 | 0.12 | 0.06 | 0.17 | 0.18 | 0.12 | 0.25 | 0.70 | 0.59 | 0.81 |
| 244.7 | 5.5 | 0.10 | 0.05 | 0.16 | 0.17 | 0.11 | 0.23 | 0.73 | 0.62 | 0.84 |
| 270.4 | 5.6 | 0.09 | 0.04 | 0.14 | 0.15 | 0.09 | 0.22 | 0.76 | 0.65 | 0.86 |
| 298.9 | 5.7 | 0.08 | 0.03 | 0.12 | 0.14 | 0.08 | 0.20 | 0.78 | 0.68 | 0.89 |
| 330.3 | 5.8 | 0.07 | 0.03 | 0.11 | 0.13 | 0.07 | 0.18 | 0.81 | 0.71 | 0.90 |
| 365.0 | 5.9 | 0.06 | 0.02 | 0.10 | 0.11 | 0.06 | 0.17 | 0.83 | 0.74 | 0.92 |
| 403.4 | 6.0 | 0.05 | 0.02 | 0.09 | 0.10 | 0.05 | 0.15 | 0.85 | 0.76 | 0.93 |

**Additional file 1**

Probability of liver steatosis as detected by the natural logarithm of the lipid accumulation product in males and females. Abbreviations; LAP = lipid accumulation product; lnLAP = natural logarithm of LAP; Prob = probability; Lower = lower 95% confidence interval; Upper = upper 95% confidence interval.
